# N6-METHYLDEOXYADENOSINE (N6MEDA) IS ASSOCIATED WITH AGING

**DOI:** 10.1093/geroni/igae098.0737

**Published:** 2024-12-31

**Authors:** Natalia Tretyakova

**Affiliations:** University of Minnesota, Minneapolis, Minnesota, United States

## Abstract

N6-methyldeoxyadenosine (N6medA) is a recently discovered DNA mark that has been proposed to be involved in epigenetic regulation, DNA repair, and memory formation. N6medA is dynamically regulated in the mouse brain following environmental stress and plays a role in the formation of fear extinction memories, immune response, and in neuroblastoma. However, the biological roles of N6medA as an epigenetic mark have been questioned due to it extremely low abundance in the genome and conflicting information regarding its origins. We have developed an ultrasensitive isotope dilution nanoLC-methodology for N6medA in genomic DNA and investigated the potential roles of N6medA in human aging and in Alzheimer’s Disease (AD). We found that the global genomic N6medA in prefrontal cortex is positively correlated with human’s chronological age, and there is an increasing trend for N6medA levels in mild cognitive impairment and Alzheimer’s Disease. Genome wide mapping of N6medA in DNA isolated from human prefrontal cortex indicated that increased adenine methylation in AD was preferentially found in genes associated with neurological function and neurological diseases. Mass spectrometry-based affinity proteomics experiments identified several protein readers of N6medA as well as proteins that are prevented from binding to DNA in the presence of methylated adenine. Taken together, our results suggest that N6medA mediates protein binding to DNA, accumulates in genomic DNA of the brain during aging and could contribute to the development of Alzheimer’s Disease

